# Q10 supplementation effects on cardiac enzyme CK-MB and troponin in patients undergoing coronary artery bypass graft: a randomized, double-blinded, placebo-controlled clinical trial

**DOI:** 10.15171/jcvtr.2016.01

**Published:** 2016-03-14

**Authors:** Jalal Moludi, Seyedali Keshavarz, Ali Sadeghpour Tabaee, Saeid Safiri, Reza Pakzad

**Affiliations:** ^1^Department of Biochemistry and Diet Therapy, Faculty of Nutrition, Nutrition Research Center, Tabriz University of Medical Sciences, Tabriz, Iran; ^2^School of Nutritional Sciences and Dietetics, Tehran University of Medical Sciences, Tehran, Iran; ^3^Rajaie Cardiovascular Medical and Research Center, Iran University of Medical Sciences, Tehran, Iran; ^4^Managerial Epidemiology Research Center, Department of Public Health, School of Nursing and Midwifery, Maragheh University of Medical Sciences, Maragheh, Iran; ^5^Department of Epidemiology and Biostatistics, School of Public Health, Tehran University of Medical Sciences, Tehran, Iran

**Keywords:** CK-MB, Troponin, Coronary Artery Bypass, CO Q10

## Abstract

***Introduction:*** Coronary artery bypass surgery (CABG) is associated with ischemia-reperfusion injury and tissue damage. CoQ10 as an antioxidant has an important role and may have cardio-protective effects after myocardial dysfunction and CABG. We aimed to evaluate whether CoQ10 has a myocardial cardio protective impact on cardiac biomarkers after CABG.

***Methods:*** In this double-blind study, 80 patients with coronary artery disease (CAD) who underwent CABG surgery were divided into intervention and control groups and received Q10 supplement or placebo, respectively. The surgical characteristics of the patients in the two groups were similar. The intervention group received 150 mg of Q10 supplement per day for 7 days before the surgery. The control group received placebo capsule. After operation the inter- and intra-group blood levels of CK-MB and troponin, before and after supplementation and 12 hours after the CABG, and postoperative outcomes such as intensive care unit (ICU) stay and hospital stay were compared.

***Results:*** In this study, 40 subjects were located in each group. The participation rate was 97.5% and men and women accounted for 52.5% and 47.5% respectively. The mean age of the subjects was 58.17 ± 8.55. The two groups were not significantly different in terms of basic variables. Within-group comparison showed a significant increase in the level of troponin enzymes over time (*P* < 0.001) and CK-MB (*P* < 0.001). However, between-group comparison showed no significant difference between the two groups in terms of CK-MB (*P* = 0.384) and troponin (*P* = 0.115). In the end, no interaction was observed between the intervention and time on CK-MB (P = 0.095) and troponin (*P* = 0.198) variables.

***Conclusion:*** Q10 supplementation 7 days before surgery was not effective in reducing CK-MB and troponin after CABG.

## Introduction

Coronary artery bypass surgery (CABG) surgery is a high-risk procedure. Evidence indicates that cardiopulmonary bypass (CPB) leads to inflammatory response that may be responsible for some of the major complications after open-heart surgery.^1,2^ Under reoxygenation condition, the blood flows back into the ischemic area after the surgery followed by the production of free radicals (e.g. superoxide anion) disrupting the production of ATP.^3^ Metabolic changes that occur during ischemia and cardiac myocytes, reduce glutathione system in the first line of defense against ROS in mitochondria. Reduced glutathione levels lead to the formation of ROS, oxidative stress, and increase intracellular calcium levels.^4^ Dysrhythmia after the surgery, as an indicator visible from oxidant stress during operation is considered, and can provide a basis for the comparison of strategies in protecting the heart during cardiac surgery.^5^ CK-MB and Troponin as a marker of myocardial infarction are used to measure these biomarkers to assess patients and controls in terms of infarction after the surgery.^6^ According to previous studies, a low level of troponin after the surgery is predictive of success in short and long-term. For this reason, decreases in the levels of these biomarkers could be useful.^7^ Peak plasma levels of troponin in the study of Cummins B, and others, was expressed 14 hours after the onset of acute myocardial infarction (AMI). For this reason, we also measured troponin in the first 12 hours after the surgery.^8^ It appears that during CABG, total antioxidant capacity is significantly reduced. Coenzyme Q10, as an antioxidant can reduce free radicals and may play an effective role in reducing cardiac enzymes and complications after the surgery.^9-11^ The main objective of this study was to determine the effect of Q10 supplements on the level of CK-MB and troponin levels after elective coronary artery bypass graft.

## Materials and methods

### Design and setting

This was a double-blind randomized clinical trial implemented in Shahid Rajai Heart Center in 2013 through a parallel design.

### Sample size

Considering the type of analysis, effect size = 0.25, power = 0.80, α = 0.05 and the fact that the two groups were studied the total number of required samples was determined 82 using the software G-power.^12^

### Inclusion and exclusion criteria

All patients who qualified elective CABG surgery based on angiography were included in the study. Also, patients who have experience of warfarin use, antioxidant intake, hybrid surgery, creatinine greater than 1.5 and advance heart failure were excluded from the present study.

### Sampling and random assignment

**Figure 1:**
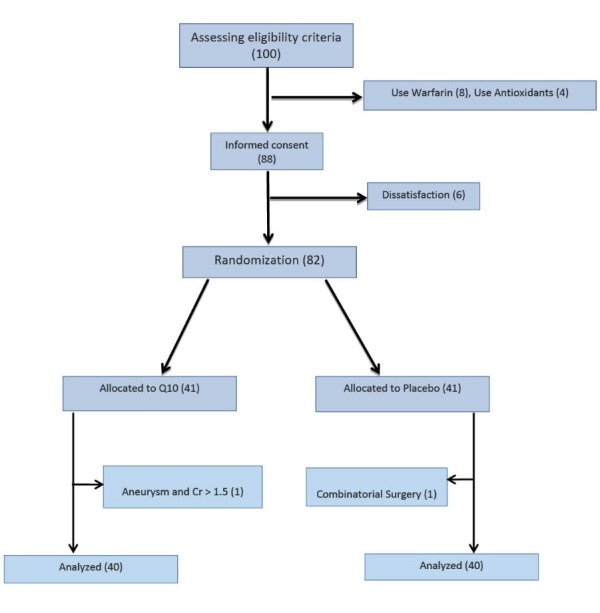
Flowchart of study design.

At first, 100 patients candidate for CABG surgery admitted to hospital were selected with a daily visit to the different wards of Shahid Rajai hospital and according to the study inclusion and exclusion criteria, 88 of them were selected and given a full explanation about the goals of the study and were asked to offer informed consent. Six people refused to consent after hearing explanations and were excluded from the study (Figure 1). Finally, 82 patients were assigned to experimental and control groups using randomized block method in size of 4. To create random blocks, RAS (Research Analysis and Statistics) software was used.

### Intervention and blinding

The considered intervention was a supplement called Q10 which was a vitamin-like enzyme and can reduce the CK-MB and troponin enzymes. This supplement was made in 50-mg capsules by the Elixir Pharmaceuticals, Inc. Eighty-two boxes were used in this study and 41 of which had Label A and contained 50 mg Q10 supplement capsules and 41 other capsules had Label B and contained similar and empty capsules as placebo which were made by the same company. After determining patients and arranging blocks, boxes were given to patients and given that the study was double blinded, patient and the nurse who was the responsible for the hospital ward were unaware from the contents of the boxes and capsules.

For the intervention group, Q10 supplement was daily given to patients as 50-mg capsules for seven days and the control group was given capsules as placebo with identical shape and without any drug content (only a similar coating).

### Procedure

Standard intravenous anesthesia (with 10 mg oral lorazepam) was done. Similar standard surgical techniques was done in all cases. Sternotomy was performed from the middle part of the sternum. In addition, grafting one of internal thoracic arteries with 1-5 peripheral veins from lower limbs was considered in each case. CPB was prepared and an anstomosised with proximal area with moderate hypothermia. After surgery, patients were transferred to the intensive care unit (ICU) and separated from mechanical ventilation with the following criteria: Hemodynamic stability, ambient temperature over 36°C, cooperation, lack of major bleeding and acceptable arterial blood gases.

### Measurements

The variables of age, gender, average graft time, average time of cross clamp and graft and pre-operation ejection fraction were measured as basic variables. Venous blood samples were taken from the antecubital without tourniquet for cardiac enzyme levels. For the measurement of outcome variables such as CK-MB and Troponin (By chemiluminescence: bioMérieux launches VIDAS^®^ Troponin I Ultra kit is made in France and the MB kit Audit Diagnostics facility in Cork, Ireland), blood samples of all the patients were measured in terms of these enzymes in three occasions before beginning supplements and seven days after supplementation and twelve hours after surgery and were compared on this basis. All patients were transferred to ICU after surgery.

### Statistical analysis

Data were analyzed using SPSS version 16. To study the normality of data distribution, Kolmogorov-Smirnov test was used. For comparing baseline data, *t* test and chi-square tests were used and to compare the effect of supplementation during the three occasions one-way repeated measure ANOVA was used. Moreover, Bonferroni test was used for pair-wise comparisons. Data are expressed as mean ± SD for the quantitative and No. (%) for the categorical variables. Significance level was set at 0.05.

## Results

### Comparing baseline data on two groups

Given that after random allocation of subjects in the intervention group, one patient experienced Cr >1.5 and the aneurysm. Besides, one patient in the control group was excluded because he/she experienced combinatorial surgery. Finally, 40 patients were compared in each group. Participant rate was 97.5% and no side effects were observed. All the information was measured and there was no missing in this regard. All quantitative variables were normal. The mean age of the subjects was 58.17 (SD = 8.55, range = 33-70). Forty-two patients were male (52.5%) and 38 patients (47.5%) were female. Comparing baseline variables between the two groups of intervention and control showed no significant difference of variable age between the two groups (*P*=0.0836). Variable distribution of blood pressure (*P*=0.115), sex (*P*=0.179) and diabetes (*P*=0.799) were not significantly different between the two groups. Besides, the variables bypass time (*P*=0.667), cross clamp time (*P*=0.621), Pre-operation (ejection fraction) (*P*=0.269) and graft (*P*=0.179) were not significantly different between the two groups (Table 1).

**Table 1:** Comparison of basic variables between the two groups^a^

**Variable**	**Intervention (n= 40)**	**Control (n= 40)**	**P-Value**
Age	58.37± 1.25	57.97± 1.45	0.0836
Bypass Time	73.67± 22.13	71.3 ± 20.8	0.667
Cross Clamp time	37 ± 13.15	38 ± 15.15	0.621
Graft	3.27±0.84	3.02±0.80	0.179
Pre-operation ejection fraction	36.7±8.8	34.7±7.07	0.269
Hypertension			0.115
Yes	21 (60)	14 (40)	
No	19 (42.2)	26 (57.8)	
Sex			0.179
Male	24 (57.1)	18 (42.9)	
Female	16 (42.1)	22 (59.9)	
Diabetes Melitus			0.799
Yes	11 (52.4)	10 (47.6)	
No	29 (48.2)	30 (50.8)	

^a^Quantitative and qualitative variables were presented as mean ± SD and number (percent), respectively. Quantitative and qualitative variables were compared between two groups using independent *t* test and chi-square test, respectively. Significance level was considered as 0.05.

### Within subject for CK-MB and troponin variables

**Figure 2:**
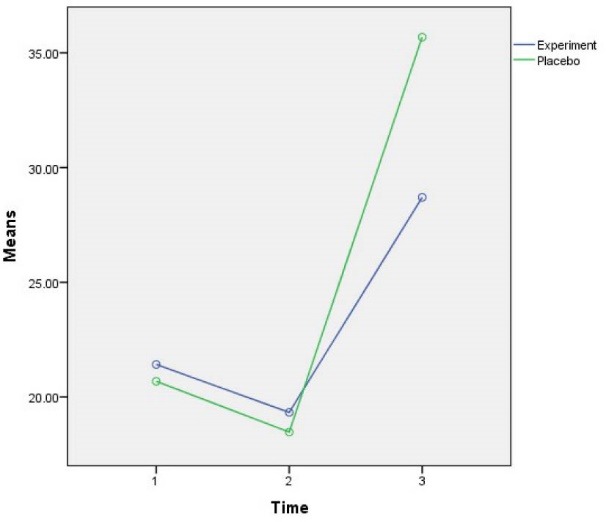
Average variable CK-MB between the intervention and control groups at three time points measured.

**Figure 3:**
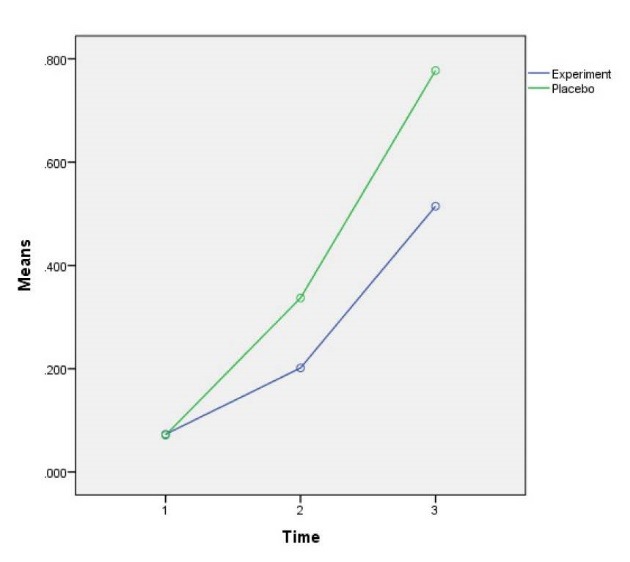
Average variable troponin between the intervention and control groups at three time points measured.

Comparing the average variable CK-MB in three time measurements showed that the average variable CK-MB decreased over time and then increased so that it was (21.04 ± 8.33) at the first time, (18.89 ± 6.71) at the second time, and (32.19 ± 20.83) at the third time and the three values were significantly different (*P*<0.001). Bonferroni test results used for pair-wise comparison showed that CK-MB variable at the second time is less than the first time (*P*<0.001). Moreover, at the third time it is more than the first time (*P*<0.001) and at the third time it is more than the second time (*P*<0.001) and all of these cases were significant (Table 2 and Figure 2). Average variable troponin in 3 measurements showed that the average variable troponin increased over time so that it was (0.072 ± 0.01) at the first time, (0.26 ± 0.48) at the second time and (0.64 ± 0.76) at the third time, and the difference between these three values was significantly different ​​together (*P*<0.001). Bonferroni test results showed that troponin variable at the second time is more than the first time (*P*<0.001), at the third time is more than the second time (*P*<0.001) and at the third time is more than the second time (*P*<0.001), which all were significant (Table 2 and Figure 3).

**Table 2:** Comparing the CK-MB and troponin levels between two study groups

**Variable**	**Time 1**	**Time 2**	**Time 3**	**Total**		**Between-subject**	**Within-subject**	Interaction Intervention*Time
				**Intervention (40)**	**Control (40)**			
CK-MB	21.04 ± 8.33	18.89 ± 6.71	32.19 ± 20.83	23.14±1.45	24.94±1.45	F= 0.767 df= 1, 78 *P*=0.384	#F = 28.04‏ df= 1.12, 87.82 *P* < 0.001	F=28.04 df=1.12, 1.12 *P*= 0.095
Troponin	0.072 ± 0.1	0.26 ± 0.48	0.64 ± 0.76	0.263± 0.059	0.395 ± 0.059	F=2.544 df=1,78 *P*=0.115	*F = 32. 40‏ df= 1.64, 128.07 *P* < 0.001	F=1.66 df=1.64, 1.64 *P*=0.198

The mean±SD studied variables were presented in three time points. ^#^Between and within subjects analyses were done using Greenhouse-Geisser test. *Significance level was considered as 0.05.

### Intervention (between subject variable) for CK-MB and troponin

Although the average variable CK-MB and troponin in the intervention group was less than the control group, but these differences were not significant in both groups (*P*=0.115, *P*=0.384) (Table 2).

### Interaction between the time and intervention in CK-MB and Troponin variables

The results showed that the interaction between time and intervention for CK-MB (*P*=0.095) and troponin (*P*=0.198) variables do not exist (Table 2).

## Discussion

The present clinical trial was performed to study the effects of oral CoQ10 supplements before surgery to increase the level of CK-MB and troponin I in patients undergoing CABG. In the present study, increased serum concentrations of CK-MB and troponin levels in both Q10 groups and the placebo group was observed. However, significant differences were not observed over time.

There is a lot of information, which suggests that protecting heart during CABG has important long-term effects after surgery.^13,14^ Cardioplegia by ischemia-reperfusion injury (IRI, #5) has harmful effects on the heart^15^ which may be prevented by antioxidant effects caused by supplements,^16^ reactive oxygen intermediates can cause imbalance and excessive intracellular calcium and these states show themselves with increasing levels of cardiac enzymes of CK-MB and troponin.^17^

Interventional studies of coronary artery showed that the peak level of CK-MB not only is along with higher mortality, but with a higher risk of events after surgery leading to further cost of treatment in these patients.^18-20^ However, trying to identify a threshold and the upper and lower limits that predict patients’ prognosis is faced with failure.^21^

According to the definition of ACC, cardiac biomarkers level (CK-MB and troponin I) is considered as more than 3 times the upper limit of normal as myocardial infarction and the range between one and three times is considered as myocardial injury.^22^ It is surely clear that the risk of side effects increases with any increase in CK-MB. Therefore, measurement of cardiac enzymes following coronary intervention is strongly recommended.^23^ and the reduced level of cardiac enzymes is along with improved surgical outcomes in the short-term and long-term,^24-26^ although 0.1 ng/mL troponin was used as the cutoff. Even low levels may cause myocardial injury.^27^ In this study, 0.1 ng troponins were considered as high levels (positive troponin). After CBP, troponin and CKMB levels begin to rise after six hours and two hours respectively,^28^ so we used both markers.

CoQ10 plays an important role in stabilizing the membrane while helping replenish ATP storage inside the cell.^29^ these mechanisms minimize cellular damage and a decrease in cardiac enzymes can be experienced. According to previous studies,^30-32^ the used dose was determined as 150 mg per day for seven days. On the other hand, according to the study, it is found that a daily dose more than 300 mg creates negative effects such as increased levels of serum LDH.^33^ that’s why some studies that have used excessive doses of supplements have not reported a positive effect in patients.

Various studies have indicated the effect of antioxidants in reducing cardiac enzymes level^16,30^ so that Foroughinia et al study showed omega-3 positive impact in reducing the levels of cardiac enzymes.^34^ Makhija et al showed that preoperative use of Q10 can lower levels of cardiac enzymes.^11^ Other studies have shown that Q10 supplement is effective on troponin and CK-MB enzymes after surgery, even when supplement is administered only once.^32^ Pepping noted in his study that it takes at least four days to achieve therapeutic level.^35^ That is why we raised supplementation period to seven days in this study. In a review study by Bulatao and Banez,^31^ it was shown that oral supplement Q10 in spite of providing lower surgery indices including less drainage, less hospital stay and less need for inotropic had no effect on cardiac biomarkers level. Increasing the number of grafts is directly correlated with higher levels of the enzyme. Jang et al showed in his study that increasing troponin serum levels is associated with the number of involved arteries that underwent PCI.^36^ In our study, a statistically significant difference was not observed in number of grafts. Previous studies have indicated a strong correlation between the number of grafts and graft type with increased levels of cardiac enzymes as observed in one of the studies^37^ that internal mammary artery (IMA, #32) graft was along with less increase in cardiac enzyme levels. Age and gender were also evaluated as a risk factor for elevating troponin serum levels. But no significant statistical difference was observed between the two groups, age and sex with troponin. Wiviott et al in 2004 compared the effects of gender on designed cardiac enzyme levels. It was observed that the level of CK-MB and troponin is higher in men, while inflammatory marker following AMI and angina is higher in women.^38^

In this study from among 80 cases of intervention and control at baseline, 71.3% had negative troponin (troponin larger than 0.1) and 28.7% had positive troponin and after surgery, total troponin level was 25% percnt negative and 75% positive. Iner and intra group comparisons showed that Q10 supplements could not have a significant effect in reducing the levels of troponin. Although in previous studies, using some supplements such as omega-3 could have a positive effect on the number of positive troponins.^39^ In explaining why the supplement could not reduce the enzyme levels, items such as long time surgery can be cited. Because in this study, the time of surgery was more than 73 minutes, while in other studies^40^ it has been for about 49 minutes and it is obvious that whatever this period is more, the cardiac enzymes CK-MB and troponin will be higher.^41^ That is why the present study has not been able to be successful in reducing the level of cardiac enzymes.

After surgery, the incidence of arrhythmia in the intervention group who received CoQ10 supplement was significantly lower compared with the control group. However, hospital stay and ICU stay were not significantly different between the two groups. Although in some studies, the effect of supplement was seen to reduce the length of stay in the ICU and reduced drainage.^42,43^

According to the results obtained from a randomized double-blinded study, the prescription of Q10 supplements was not effective unlike the designers’ original idea in reducing cardiac enzyme (CK-MB) and troponin after CABG.

## Limitations of the study

The results of the present study were based on a research on a small population of patients undergoing coronary bypass surgery that may be the main reason for not observing the effect of supplementation in reducing cardiac enzyme levels. Besides, the short time of supplementation or inadequate dose of Q10 may have a significant role in the lack of impact of reducing troponin. Moreover the limitations of this study include lack of body antioxidant levels during the period mentioned, in fact, our blindfold, just looked at the effect of antioxidant supplements That’s why more studies are needed in this regard.

## Acknowledgments

We would like to offer our appreciation and thanks to Tehran Rajaee heart hospital nurses and doctors who have supported us.

## Ethical issues

After obtaining the ethics code of Tehran University of Medical Sciences (25555-161-01-93) and registration at Iranian Registry of Clinical Trials (IRCT ID: 2013122815957N1) was conducted according to the Helsinki protocol.

## Competing interests

None**.**
